# A phase I study to assess afatinib in combination with carboplatin or with carboplatin plus paclitaxel in patients with advanced solid tumors

**DOI:** 10.1007/s00280-018-3661-1

**Published:** 2018-08-07

**Authors:** Mary E. R. O’Brien, Debashis Sarker, Jaishree Bhosle, Kiruthikah Thillai, Timothy A. Yap, Martina Uttenreuther-Fischer, Karine Pemberton, Xidong Jin, Sabrina Wiebe, Johann de Bono, James Spicer

**Affiliations:** 1 Royal Marsden NHS Foundation Trust, Downs Road, Sutton, UK; 2 King’s College London, Guy’s Hospital, 3rd Floor Bermondsey Wing, London, UK; 3 Boehringer Ingelheim International GmbH, Biberach, Germany; 4 Boehringer Ingelheim Ltd, Bracknell, UK; 5 Boehringer Ingelheim Pharmaceuticals, Inc., Ridgefield, CT USA

**Keywords:** Afatinib, Carboplatin, Paclitaxel, Phase I, Solid tumors

## Abstract

**Purpose:**

Afatinib, an irreversible ErbB family blocker, has demonstrated preclinical antitumor activity with chemotherapy.

**Methods:**

As part of a phase I trial in patients with advanced solid tumors (NCT00809133; 3 + 3 dose-escalation design), we determined the maximum tolerated dose (MTD) of afatinib with carboplatin (A/C) or with carboplatin plus paclitaxel (A/C/P). Starting doses: afatinib 20 mg/day, carboplatin AUC6 (A/C) with paclitaxel 175 mg/m^2^ (A/C/P) (chemotherapy: Day 1 of 21-day cycles). The primary objective was to determine the MTDs; safety, pharmacokinetics and antitumor activity were also evaluated.

**Results:**

Thirty-eight patients received A/C (*n* = 12) or A/C/P (*n* = 26). No dose-limiting toxicities (DLTs) were reported with A(20 mg)/C(AUC6). One patient experienced DLT in the A(40 mg)/C(AUC6) cohort (grade 3 acneiform rash); A(40 mg)/C(AUC6) was determined as the recommended phase II dose (RP2D) for A/C. Two patients each had DLTs with A(20 mg/day)/C(AUC6)/P(175 mg/m^2^): fatigue, infection, diarrhea, small intestine hemorrhage, dehydration, renal impairment, neutropenic sepsis (*n* = 1), mucositis (*n* = 1); A(40 mg)/C(AUC5)/P(175 mg/m^2^): febrile neutropenia (*n* = 1), mucositis, fatigue (*n* = 1); and A(30 mg)/C(AUC5)/P(175 mg/m^2^): stomatitis (*n* = 1), mucositis (*n* = 1). No DLT was observed with A(20 mg)/C(AUC5)/P(175 mg/m^2^), determined as the RP2D for A/C/P. The most frequent drug-related adverse events were (A/C; A/C/P): rash (75%; 73%), fatigue (67%; 69%), and diarrhea (58%; 88%). Drug plasma concentrations were similar between cycles, suggesting no drug–drug interactions. Objective response rates in these heavily pretreated patients were A/C, 3/12 (25%); A/C/P, 5/26 (19%).

**Conclusions:**

Afatinib 40 mg/day (approved monotherapy dose) with carboplatin AUC6, and afatinib 20 mg/day with carboplatin AUC5 and paclitaxel 175 mg/m^2^ demonstrated manageable safety and antitumor activity. Afatinib > 20 mg/day in the triple combination was not well tolerated.

## Introduction

Afatinib is an irreversible ErbB family blocker, which binds covalently to the intracellular kinase domains of, and prevents signaling from, all kinase-active members of the ErbB family. It is, therefore, active against epidermal growth factor receptor (EGFR), human epidermal growth factor receptor 2 (HER2) and ErbB4, and indirectly inhibits transphosphorylation of the kinase-inactive ErbB3 [1]. As a single agent, afatinib is approved for the first-line treatment of advanced *EGFR* mutation-positive lung adenocarcinoma, having demonstrated improved progression-free survival (PFS) versus platinum-based chemotherapy in these patients [2, 3]. In addition, afatinib was recently approved for the treatment of patients unselected for *EGFR* mutations with advanced squamous cell carcinoma of the lung following first-line chemotherapy, with activity superior to erlotinib in this setting [4].

Given their non-overlapping mechanisms of action, the addition of targeted therapies such as afatinib to existing chemotherapy regimens may improve outcomes in patients with advanced solid tumors. In vitro cell-based assays have shown greater efficacy with afatinib combined with paclitaxel than with single-agent treatment [5]. The combination of afatinib with paclitaxel or docetaxel in xenograft animal models has also demonstrated improved efficacy compared with single-agent therapy [6].

The clinical feasibility and tolerability of combining afatinib with paclitaxel, and with paclitaxel plus bevacizumab, were previously explored in earlier parts of this trial (a total of four drug combinations were assessed in patients with advanced solid tumors in this trial [NCT00809133]) [5, 7]. Both of these earlier combination regimens demonstrated manageable safety profiles and antitumor activity at the maximum tolerated doses (MTDs). Here, we evaluated the combination of afatinib with carboplatin, and with carboplatin and paclitaxel, in patients with advanced solid tumors.

## Materials and methods

### Patients

Patients eligible for treatment had advanced, non-resectable and/or metastatic solid tumors suitable for treatment with either carboplatin or carboplatin and paclitaxel, and were recruited at two centers in the United Kingdom. Key eligibility criteria included age ≥ 18 years; Eastern Cooperative Oncology Group performance status (ECOG PS) of 0 or 1; life expectancy of at least 3 months and adequate organ function (cardiac left ventricular function with resting ejection fraction [LVEF] ≥ 50%; absolute neutrophil count ≥ 1.5 × 10^9^/L [> 2.0 × 10^9^/L for patients allocated to carboplatin]; platelets ≥ 100,000/µL; total bilirubin ≤ 26 µmol/L; aspartate aminotransferase and/or alanine aminotransferase ≤ 2.5 times the upper limit of normal; creatinine ≤ 132 µmol/L and creatinine clearance > 60 mL/min by Cockcroft–Gault equation).

Exclusion criteria included gastrointestinal tract disease that could impair drug absorption; significant cardiovascular disease; active infectious disease; known interstitial lung disease; untreated or symptomatic brain metastases; persistent grade ≥ 2 neuropathy or neurotoxicity from any cause; and treatment with chemotherapy, immunotherapy, radiotherapy, biologic therapy, hormone therapy, EGFR- or HER2-targeting drugs or other investigational drugs within 4 weeks prior to starting trial medication.

The study was conducted in accordance with the Declaration of Helsinki and Good Clinical Practice, and ethical approval was provided by the UK Integrated Research Application System. All patients provided written informed consent.

### Study design and treatment

A phase I open-label trial assessed four different drug combinations using a 3 + 3 dose-escalation design (NCT00809133); previous analyses of other combination regimens included in the trial have been reported [5, 7]. In this report, afatinib was evaluated in combination with carboplatin (A/C) or carboplatin plus paclitaxel (A/C/P). In both combinations, oral afatinib was administered once daily, beginning on Day 2 of Cycle 1 in 21-day cycles, at a starting dose of 20 mg/day, and escalated to 40 mg (the approved monotherapy starting dose) and then 50 mg in subsequent dose cohorts. Chemotherapy was administered intravenously on Day 1 of each 21-day cycle: carboplatin at a dose targeting an area under the concentration–time curve of 6 mg/mL.min (AUC6), and paclitaxel at 175 mg/m^2^ (carboplatin was administered after paclitaxel, consistent with standard medical practice in the United Kingdom) [8]. Target doses for carboplatin were calculated using the Calvert formula (dose [mg] = target AUC [mg/mL.min] × (glomerular filtration rate [GFR; mL/min] + 25 mL/min)). Based on the American Society for Clinical Oncology guidelines, the GFR used in the Calvert formula was capped at 125 mL/min [9]. In the event of toxicity with carboplatin AUC6, a dose reduction to AUC5 was allowed. Patients continued combination treatment for six cycles or until tumor progression, intolerable adverse events (AEs) or withdrawal of consent. Patients with clinical benefit after six cycles could continue either with combination treatment or single-agent afatinib.

The primary endpoint was safety, assessed as dose-limiting toxicities (DLTs) to define the MTD of the two afatinib–chemotherapy combinations. MTD was defined as the highest dose of afatinib in combination with carboplatin, or with carboplatin and paclitaxel, at which no more than one of six patients experienced a DLT during Cycle 1. To be evaluable for determination of the MTD, patients must have completed the first 2 weeks of therapy or have experienced a DLT; patients who did not meet either of these criteria were replaced within the respective dose cohort. Secondary endpoints included pharmacokinetic parameters and antitumor activity (objective tumor responses [OR]).

### Assessments

All patients were assessed for AEs according to the National Cancer Institute Common Terminology Criteria for Adverse Events (NCI CTCAE, version 3.0); relationship to study treatment was assessed by the investigators.

DLTs were defined as any of the following drug-related AEs: grade 4 uncomplicated neutropenia (i.e., fever ≤ 38.3 °C) for > 7 days; neutropenia associated with fever > 38.5 °C; platelets < 25 × 10^9^/L or grade 3 thrombocytopenia associated with bleeding requiring transfusion; grade ≥ 2 decrease in LVEF; uncontrolled hypertension despite multiple anti-hypertension therapies; grade ≥ 2 worsening of renal function; grade > 2 diarrhea despite anti-diarrheal treatment; persistent grade ≥ 2 diarrhea for ≥ 7 days despite supportive care; grade > 2 nausea and/or vomiting despite antiemetic treatment; persistent grade ≥ 2 vomiting for ≥ 7 days despite supportive care; and all other non-hematologic toxicities of grade ≥ 3, except incompletely treated nausea, vomiting or diarrhea.

For the assessment of pharmacokinetic parameters of afatinib (AUC_τ,ss_ and *C*_max,ss_), total platinum (AUC_0−24_ and *C*_max_) and paclitaxel (AUC_0−23_ and *C*_max_), blood was collected on Days 1 and 2 of Cycles 1 and 2. Samples were taken pre- and post- (1.0, 1.5, 2.0, 3.0, 4.0, 6.0, 8.0 and 24.0 h) carboplatin infusion (in the A/C arm) and pre- and post- (1.0, 2.0, 3.0, 3.5, 4.0, 4.5, 5.0, 6.0, 8.0, 9.0 and 24.0 h) paclitaxel infusion (in the A/C/P arm). Of note, the 1.0- and 2.0-h time points post-paclitaxel infusion were conducted only during Cycle 2. A sample was also collected on Day 15 of Cycle 1 before the administration of afatinib in both treatment combinations. Plasma concentrations of afatinib and paclitaxel were analyzed by a validated high-performance liquid chromatography–tandem mass spectrometry method at Boehringer Ingelheim (Biberach, Germany) and Nuvisan GmbH (Neu-Ulm, Germany), respectively. Concentrations of total platinum (from carboplatin) were determined by a validated inductively coupled plasma/mass spectrometry assay at Nuvisan GmbH (Neu-Ulm, Germany).

Tumor assessments were performed by the investigators at screening and every 6 weeks after the start of treatment until progressive disease (PD) according to Response Evaluation Criteria in Solid Tumors (RECIST, version 1.0).

### Statistical analysis

AEs and antitumor activity were assessed in all patients who received at least one dose of afatinib. All analyses in this trial were descriptive and exploratory.

## Results

### Patients and treatment exposure

Thirty-eight patients were treated in the trial, with the most common tumor type (A/C, 58%; A/C/P, 65%) being non-small cell lung cancer (NSCLC). Additional baseline demographics and disease characteristics are shown in Table 1. Twelve patients received the combination of A/C and 26 received A/C/P. In the A/C arm, 10 (83%) patients completed at least one cycle and 6 (50%) patients completed at least six cycles of treatment; all patients discontinued study treatment due to PD. In the A/C/P arm, 24 (92%) patients completed at least one cycle and 11 (42%) patients completed at least six cycles of treatment. The primary reason for termination of study treatment in the A/C/P arm was PD in 21 patients (81%) and AEs, including DLTs, in 5 patients (19%). Median treatment duration was 106 days (range 13–390) for A/C and 85 days (range 6–401) for A/C/P.

**Table 1:** Patient demographics at baseline

	A/C (*n* = 12)	A/C/P (*n* = 26)
Age, years		
Median (range)	57.5 (35–80)	60.5 (26–73)
Gender, *n* (%)		
Male	8 (67)	13 (50)
Female	4 (33)	13 (50)
Race, *n* (%)		
White	8 (67)	23 (88)
Black	2 (17)	1 (4)
Asian	2 (17)	2 (8)
ECOG PS, *n* (%)		
0	0	2 (8)
1	12 (100)	23 (88)
2	0	1 (4)^a^
Time from first histologic diagnosis, years		
Median (range)	1.5 (0.7–6.3)	1.9 (0.7–6.6)
Tumor type, *n* (%)		
NSCLC	7 (58)	17 (65)
Pancreas	1 (8)	3 (12)
Gastrointestinal tract	2 (17)	0
Breast	0	2 (8)
Other^b^	2 (17)	4 (15)
Patients with previous anti-cancer therapy, *n* (%)		
Surgery	5 (42)	11 (42)
Systemic chemotherapy	12 (100)	26 (100)
Immunotherapy	1 (8)	1 (4)
Hormone therapy	0 (0)	1 (4)
Radiotherapy	6 (50)	13 (50)
Other (including biologic therapy)	7 (58)	6 (23)

*A*/*C* afatinib plus carboplatin, *A*/*C*/*P* afatinib plus carboplatin plus paclitaxel, *ECOG PS* Eastern Cooperative Oncology Group performance status; *NSCLC* non-small cell lung cancer

^a^ECOG PS declined between screening and baseline; baseline visit was 2 days after the screening visit for this patient

^b^Esophageal (*n* = 1) and ovarian cancer (*n* = 1) in the A/C arm, adrenal (*n* = 1), biliary tree (*n* = 1), bladder (*n* = 1) and endometrial cancer (*n* = 1) in the A/C/P arm

### MTD assessment

**Fig. 1:**
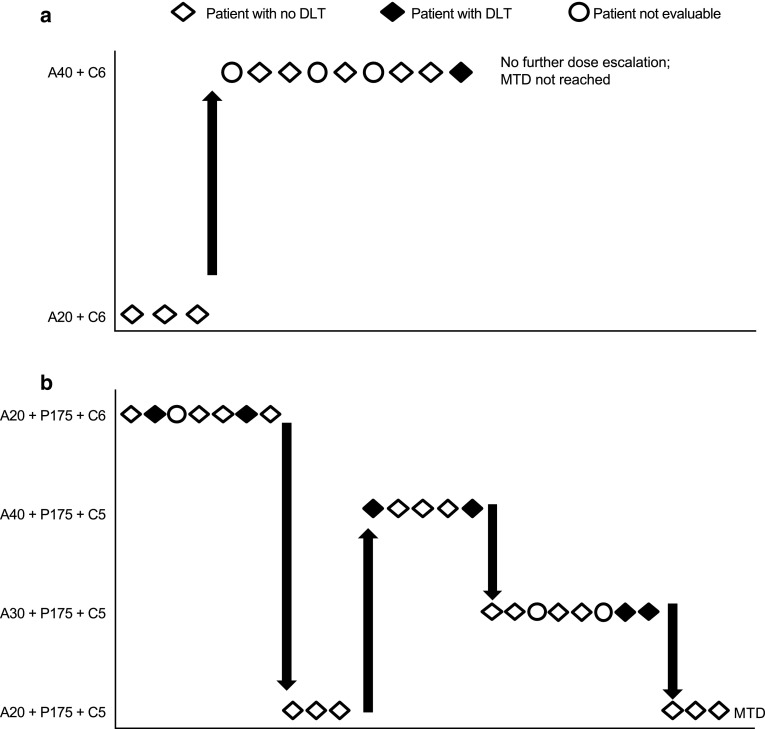
Dose-escalation schema and incidence of DLTs in the A/C arm (**a**) and the A/C/P arm (**b**). *A20* + *C6*, afatinib 20 mg/day + carboplatin AUC6; *A40* + *C6*, afatinib 40 mg/day + carboplatin AUC6; *A20* + *P175* + *C5*, afatinib 20 mg/day + paclitaxel 175 mg/m^2^ + carboplatin AUC5; *A20* + *P175* + *C6*, afatinib 20 mg/day + paclitaxel 175 mg/m^2^ + carboplatin AUC6; *A30* + *P175* + *C5*, afatinib 30 mg/day + paclitaxel 175 mg/m^2^ + carboplatin AUC5; *A40* + *P175* + *C5*, afatinib 40 mg/day + paclitaxel 175 mg/m^2^ + carboplatin AUC5

In the A/C arm, nine patients were evaluable for the determination of MTD (Fig. 1a; Table 2). In Cohort 1 (afatinib 20 mg plus carboplatin AUC6), three patients received combination treatment and no DLTs were reported. In Cohort 2 (afatinib 40 mg plus carboplatin AUC6), no DLTs were reported in the first three evaluable patients (two patients were not evaluable for MTD determination and were replaced), and the cohort was expanded to nine patients (one patient was not evaluable and was replaced). Of the six patients who were evaluable for MTD in Cohort 2, one experienced a DLT (grade 3 acneiform rash). The afatinib dose, here combined with a dose of carboplatin, was not further escalated from the standard monotherapy dose of 40 mg, consistent with standard of care. Afatinib 40 mg plus carboplatin AUC6 was defined as the recommended phase II dose (RP2D) in the A/C arm, without an MTD being reached.

**Table 2:** DLTs (related to study drug) in Cycle 1 by dose cohort

	A/C		A/C/P			
	A (20 mg) + C (AUC6) (*n* = 3)	A (40 mg) + C (AUC6) (*n* = 9)	A (20 mg) + C (AUC6) + P (175 mg/m^2^) (*n* = 7)	A (20 mg) + C (AUC5) + P (175 mg/m^2^) (*n* = 6)	A (40 mg) + C (AUC5) + P (175 mg/m^2^) (*n* = 5)	A (30 mg) + C (AUC5) + P (175 mg/m^2^) (*n* = 8)
Patients evaluable for MTD	3	6	6	6	5	6
Patients with a DLT	0	1	2	0	2	2
DLTs leading to permanent discontinuation of study treatment	0	0	2	0	0	0
DLTs, *n*						
Acneiform rash^a^	0	1	0	0	0	0
Mucositis^b^	0	0	1	0	1	1
Fatigue	0	0	1	0	1	0
Infection	0	0	1	0	0	0
Dehydration	0	0	1	0	0	0
Diarrhea	0	0	1	0	0	0
Febrile neutropenia/neutropenic sepsis^d^	0	0	1	0	1	0
Renal impairment	0	0	1	0	0	0
Small intestinal hemorrhage	0	0	1	0	0	0
Stomatitis	0	0	0	0	0	1

*A*/*C* afatinib plus carboplatin, *A*/*C*/*P* afatinib plus carboplatin plus paclitaxel, *AUC5* area under the concentration–time curve of 5 mg/mL min, *AUC6* area under the concentration–time curve of 6 mg/mL min, *DLT* dose-limiting toxicity, *MTD* maximum tolerated dose

^a^Preferred term: dermatitis acneiform

^b^Preferred term: mucosal inflammation

^c^ *Clostridium difficile*

^d^Includes one patient with febrile neutropenia and one patient with neutropenic sepsis

In the A/C/P arm, 23 of 26 patients were evaluable for assessment of the MTD (Fig. 1b; Table 2). Of the seven patients who received treatment in Cohort 1 (afatinib 20 mg plus carboplatin AUC6 plus paclitaxel 175 mg/m^2^), six were evaluable for MTD and two had DLTs, which included grade 3 neutropenic sepsis with *Clostridium difficile* diarrhea in one patient and grade 3 mucositis in the other patient (Table 2). The carboplatin dose was decreased to AUC5 in Cohort 2 (afatinib 20 mg plus carboplatin AUC5 plus paclitaxel 175 mg/m^2^), in which three patients received treatment and no DLTs were reported. The afatinib dose was then increased to 40 mg in Cohort 3 (afatinib 40 mg plus carboplatin AUC5 plus paclitaxel 175 mg/m^2^), and five patients received treatment and were evaluable for MTD; two patients had DLTs (grade 3 fatigue and grade 3 mucositis in one patient; grade 3 febrile neutropenia in another). Owing to the DLTs observed, an intermediate afatinib dose of 30 mg was explored in Cohort 4 (afatinib 30 mg plus carboplatin AUC5 plus paclitaxel 175 mg/m^2^). Eight patients received this treatment and, of the six patients evaluable for MTD, two reported DLTs (grade 3 stomatitis; grade 3 mucositis). The next lower dose level (afatinib 20 mg plus carboplatin AUC5 plus paclitaxel 175 mg/m^2^) was then expanded with three patients, in addition to the three patients already treated with these doses in Cohort 2, and no DLTs were reported. Thus, afatinib 20 mg was identified as the MTD in combination with carboplatin AUC5 and paclitaxel 175 mg/m^2^. This dose was also identified as the RP2D for the A/C/P combination.

### Adverse events

The most frequent drug-related AEs of any grade in the A/C arm were rash (grouped term; *n* = 9, 75%), which was most commonly acneiform, fatigue (*n* = 8, 67%) and diarrhea (*n* = 7, 58%). Most AEs were of CTCAE grade 1 or 2 in intensity (Table 3), and the only non-hematologic treatment-related grade 3 AEs in this arm were rash, fatigue and diarrhea (one patient each; 8%). There were no treatment-related grade ≥ 4 AEs.

**Table 3:** Treatment-related AEs in at least 10% of total patients in the A/C arm

AEs	A (20 mg) + C (AUC6) (*n* = 3), *n*		A (40 mg) + C (AUC6) (*n* = 9), *n*		Total (*n* = 12), *n* (%)	
	All grades	Grade 3^a^	All grades	Grade 3^a^	All grades	Grade 3^a^
Any AE	3	0	9	3	12 (100)	3 (25)
Rash^+^	2	0	7	1	9 (75)	1 (8)
Fatigue	3	0	5	1	8 (67)	1 (8)
Diarrhea	1	0	6	1	7 (58)	1 (8)
Nausea/vomiting^+^	1	0	5	0	6 (50)	0
Anorexia^b^	1	0	3	0	4 (33)	0
Thrombocytopenia	1	0	2	0	3 (25)	0
Epistaxis	0	0	3	0	3 (25)	0
Oropharyngeal pain	0	0	3	0	3 (25)	0
Stomatitis	0	0	2	0	2 (17)	0
Rhinitis	0	0	2	0	2 (17)	0
Taste change^c^	1	0	1	0	2 (17)	0
Dry skin	0	0	2	0	2 (17)	0
Weight loss^d^	1	0	1	0	2 (17)	0
Nasal inflammation	0	0	2	0	2 (17)	0

*A*/*C* afatinib plus carboplatin, *AE* adverse event, *AUC6* area under the concentration–time curve of 6 mg/mL min

^+^Grouped term (rash included reported preferred terms of folliculitis, cellulitis, dermatitis acneiform and rash; nausea/vomiting included reported preferred terms of nausea and vomiting)

^a^There were no treatment-related grade 4 or 5 events

^b^Preferred term: decreased appetite

^c^Preferred term: dysgeusia

^d^Preferred term: weight decreased

The most frequent drug-related AEs in the A/C/P arm were diarrhea (*n* = 23, 88%), rash (grouped term; *n* = 19, 73%) and fatigue (*n* = 18, 69%). Most AEs were of CTCAE grade 1 or 2 in intensity, and there were no grade ≥ 4 treatment-related AEs (Table 4). The most common non-hematologic treatment-related grade 3 AEs in this arm were mucositis (*n* = 4; 15%) and fatigue (*n* = 3; 12%). Seven (27%) patients, including one patient with *Clostridium difficile* infection and one patient with mucositis (discussed above as DLTs), had AEs leading to discontinuation of study treatment.

**Table 4:** Treatment-related AEs in at least 10% of total patients in the A/C/P arm

AEs	A (20 mg) + C (AUC6) + P (175 mg/m^2^) (*n* = 7), *n*		A (20 mg) + C (AUC5) + P (175 mg/m^2^) (*n* = 6), *n*		A (40 mg) + C (AUC5) + P (175 mg/m^2^) (*n* = 5), *n*		A (30 mg) + C (AUC5) + P (175 mg/m^2^) (*n* = 8), *n*		Total (*n* = 26), *n* (%)	
	All grades	Grade 3a	All grades	Grade 3a	All grades	Grade 3a	All grades	Grade 3a	All grades	Grade 3^a^
Any AE	7	3	6	2	5	4	7	3	25 (96)	12 (46)
Diarrhea	6	1	6	0	5	0	6	0	23 (88)	1 (4)
Rash^+^	2	0	5	0	5	0	7	0	19 (73)	0
Fatigue	5	1	4	0	5	2	4	0	18 (69)	3 (12)
Mucositis^b^	3	1	2	0	3	1	4	2	12 (46)	4 (15)
Anorexia^c^	4	0	1	0	3	0	2	0	10 (38)	0
Dry skin	2	0	2	0	1	0	3	0	8 (31)	0
Nausea/vomiting+	4	0	0	0	1	0	3	0	8 (31)	0
Stomatitis	0	0	3	1	3	0	1	1	7 (27)	2 (8)
Thrombocytopenia	2	2	2	2	1	0	1	0	6 (23)	4 (15)
Peripheral neuropathy	1	0	2	0	0	0	3	0	6 (23)	0
Abdominal pain	4	0	0	0	1	0	1	0	6 (23)	0
Neutropenia	1	1	3	2	1	1	0	0	5 (19)	4 (15)
Epistaxis	2	0	1	0	1	0	1	0	5 (19)	0
Nasal inflammation	0	0	2	0	3	0	0	0	5 (19)	0
Alopecia	1	0	1	0	2	0	1	0	5 (19)	0
Arthralgia	1	0	1	0	1	0	2	0	5 (19)	0
Taste change^d^	1	0	0	0	1	0	2	0	4 (15)	0
Constipation	1	0	1	0	1	0	1	0	4 (15)	0
Dyspepsia	2	0	0	0	0	0	2	0	4 (15)	0
Myalgia	1	0	3	0	0	0	0	0	4 (15)	0
Palmar–plantar erythrodysesthesia syndrome	0	0	1	0	0	0	2	0	3 (12)	0

*A*/*C*/*P* afatinib plus carboplatin plus paclitaxel, *AE* adverse event, *AUC5* area under the concentration–time curve of 5 mg/mL min, *AUC6* area under the concentration–time curve of 6 mg/mL min

^+^Grouped term (rash included reported preferred terms of rash, rash erythematous, rash pustular, dermatitis acneiform, skin fissures, blister and dermatitis; nausea/vomiting included reported preferred terms of nausea and vomiting)

^a^There were no treatment-related grade 4 or 5 events

^b^Preferred term: mucosal inflammation

^c^Preferred term: decreased appetite

^d^Preferred term: dysgeusia

### Pharmacokinetics

Table 5 summarizes key pharmacokinetic parameters for the RP2D cohorts of both treatment arms. Total platinum and paclitaxel showed a comparable exposure, based on *C*_max_ and AUC, in the absence and presence of afatinib, suggesting no clinically relevant effect of afatinib on the pharmacokinetic parameters of these drugs. Afatinib reached peak plasma concentration 1–4 h after administration in the A/C arm and 3–8 h after administration in the A/C/P arm.

**Table 5:** Pharmacokinetic parameters for carboplatin (measured as total platinum) and paclitaxel in the presence and absence of afatinib at the RP2D

	A (40 mg) + C (AUC6)				A (20 mg) + C (AUC5) + P (175 mg/m2)			
	Cycle 1 (− afatinib)		Cycle 2 (+ afatinib)		Cycle 1 (− afatinib)		Cycle 2 (+ afatinib)	
	gMean	gCV, %	gMean	gCV, %	gMean	gCV, %	gMean	gCV, %
*Afatinib*								
AUC_*Ʈ*, ss_, ng h/mL	–		*n* = 3^a^		–		*n* = 7^c^	
			465	91.8			326	60.4
*C*_max,ss_ ng/mL	–		*n* = 3		–		*n* = 7	
			44.2	9.76			18.3	52.6
*Total platinum*								
AUC_0 − 24_, ng h/mL	*n* = 9		*n* = 6^a^		*n* = 5^b^		*n* = 8^c^	
	76,800	16.9	75,700	23.6	69,700	12.4	65,400	20.9
*C*_max_. ng/mL	*n* = 9		*n* = 6		*n* = 5		*n* = 8	
	21,100	31.0	19,600	26.8	16,200	22.9	17,800	15.8
*Paclitaxel*								
AUC_0 − 23,_ ng⋅h/mL	–	–	*n* = 5^b^				*n* = 8^c^	
			10,400	21.8			10,700	32.2
*C*_max,_ ng/mL	–	–	*n* = 5				*n* = 8	
			3710	23.4			3620	50.9

*A* afatinib, *AE* adverse event, *AUC*_0−23_ area under the concentration–time curve of the analyte in plasma over 0–23 h, *AUC*_0−24_ area under the concentration–time curve of the analyte in plasma over 0–24 h, *AUC*_*Ʈ,ss*_ area under the concentration–time curve of the analyte in plasma over a dosing interval, tau, at steady state, *AUC5* area under the concentration–time curve of 5 mg/mL min, *AUC6* area under the concentration–time curve of 6 mg/mL min, *C* carboplatin, *C*_max_ maximum measured concentration of the analyte in plasma, *C*_max,ss_ *C*_max_ at steady state, *gCV* geometric coefficient of variation, *gMean* geometric mean, *P* paclitaxel, *PK* pharmacokinetic, *RP2D* recommended phase II dose

^a^Some patients who entered into the A (40 mg) + C (AUC6) cohort were not included in PK analyses in Cycle 2 due to AEs leading to treatment discontinuation, insufficient data availability for accurate PK evaluation or time violations in PK sampling

^b^One patient who entered into the A (20 mg) + C (AUC5) + P (175 mg/m^2^) cohort was not included in PK analyses in Cycle 1

^c^Additional patients who received A (30 mg) + C (AUC5) + P (175 mg/m^2^) in Cycle 1 were subsequently moved to the A (20 mg) + C (AUC5) + P (175 mg/m^2^) dose cohort for Cycle 2 and were included in the PK analyses

### Antitumor activity

In the A/C arm, three patients (25%) had a confirmed partial response (PR), five (42%) had stable disease (SD) and one (8%) had PD; three patients (25%) were not evaluable for tumor response. Five patients (42%) achieved disease control (PR or SD) for at least 6 months. No ORs were seen in the seven patients with NSCLC receiving A/C treatment, but four patients (57%) had a best response of SD, one (14%) for at least 6 months. A response duration of 10.2 months was seen in one patient with a gastrointestinal neuroendocrine tumor.

In the A/C/P arm, five patients (19%) had a confirmed PR, eleven (42%) had SD and six (23%) had PD; four patients (15%) were not evaluable for tumor response. Of the 17 patients with NSCLC treated with A/C/P, ORs were reported for three patients (18%), and eight (47%) had SD. Four patients (15%), all of whom had NSCLC, achieved disease control (PR or SD) for at least 6 months. A response duration of 10.5 months was seen in one patient with NSCLC.

## Discussion

In this phase I study, we assessed the safety and preliminary antitumor activity of afatinib in combination with carboplatin, and with carboplatin plus paclitaxel. In the A/C arm, dose escalation was not continued beyond afatinib 40 mg/day plus carboplatin AUC6 and so an MTD was not reached. The standard dose for afatinib monotherapy is 40 mg/day [10, 11]. Since manageable safety and antitumor activity were observed at this dose level with carboplatin AUC6, and no drug–drug interactions were observed, the RP2D was determined as afatinib 40 mg plus carboplatin AUC6. In the A/C/P arm, the MTD and RP2D were determined to be afatinib 20 mg plus carboplatin AUC5 plus paclitaxel 175 mg/m^2^. For this triplet combination, the afatinib single-agent dose needed to be de-escalated (from 40 to 20 mg) for the regimen to be tolerated. In both treatment combinations, afatinib at the RP2D was associated with a manageable safety profile. The most common drug-related AEs were rash, fatigue and diarrhea, and the majority of these were grade 1 or 2 in intensity. The AE profiles of the combinations were in line with the known individual safety profiles of afatinib, carboplatin and paclitaxel [5, 10, 12‒12].

Afatinib pharmacokinetic parameters were consistent with earlier studies of afatinib as a single agent [13, 14] and in combination [7], suggesting carboplatin and paclitaxel did not influence absorption and plasma concentrations of afatinib. Similarly, total platinum (from carboplatin) and paclitaxel plasma concentrations were similar in the absence and presence of afatinib, suggesting there were no relevant interactions between afatinib and paclitaxel and/or carboplatin in this study.

Evidence of antitumor activity was observed with both the A/C and A/C/P combinations. A/C was associated with disease control (PR or SD) in eight patients (67%), while disease control was observed in 16 patients (62%) receiving A/C/P. In this study, 58% and 65% of patients in the A/C and A/C/P arms, respectively, had NSCLC. Of these pretreated NSCLC patients, more than half in each treatment combination had disease control (57% in the A/C arm and 65% in the A/C/P arm), with 18% achieving an OR in the A/C/P arm. Of note, a response in one patient with NSCLC in the A/C/P combination arm was maintained for 10.5 months.

Several randomized trials have evaluated the potential clinical activity of the combination of EGFR-targeted agents with chemotherapy in advanced cancers, particularly NSCLC. Phase III studies in the first-line NSCLC setting in populations unselected for *EGFR* mutation have shown varying degrees of clinical efficacy for EGFR-targeted agent–chemotherapy combinations versus chemotherapy alone [15‒17]. In the phase III TRIBUTE trial, 1059 treatment-naïve patients with NSCLC were randomized to first-line carboplatin and paclitaxel combined with either erlotinib or placebo. No survival benefit was observed with erlotinib plus chemotherapy versus placebo plus chemotherapy in the overall trial population; however, a substantial prolongation of survival was observed in the ‘never smoked’ subgroup (22.5 vs 10.1 months; hazard ratio [HR] 0.49, 95% confidence interval [CI] 0.28–0.85; *p* = 0.01) [15]. Similarly, in the phase III TALENT trial, 1172 treatment-naïve patients with NSCLC were randomized to first-line gemcitabine and cisplatin combined with either erlotinib or placebo, with no survival benefit observed in the erlotinib group. In a small subgroup of patients who ‘never smoked’, overall survival (OS) and PFS were increased with chemotherapy and erlotinib, compared to chemotherapy alone [16]. In contrast to the TALENT and TRIBUTE studies, the phase III FAST-ACT2 trial, conducted primarily in Asia, demonstrated significantly improved PFS (HR 0.57, 95% CI 0.47–0.69; *p* < 0.0001) and OS (HR 0.79, 95% CI 0.64–0.99; *p* = 0.0420) with first-line chemotherapy (gemcitabine and platinum; Days 1 and 8 of a 4-week cycle) plus erlotinib (Days 15–28 of each cycle) over chemotherapy alone in 451 treatment-naïve patients with NSCLC. Benefit was primarily shown in those with *EGFR* mutation-positive disease; however, tumor samples were available in only 53% of the intent-to-treat population [17].

The combination of EGFR-targeted agents with chemotherapy has also been explored in the relapsed/refractory NSCLC setting. A randomized, phase II study in 165 patients with non-squamous NSCLC previously treated with one prior platinum-based chemotherapy regimen showed that pemetrexed plus erlotinib significantly improved PFS, OS and time to treatment failure versus pemetrexed alone; however, the combination was associated with an increase in grade 3/4 AEs [18]. We have previously reported on the combination of afatinib and weekly paclitaxel in patients with advanced solid tumors [5]. This regimen has since been assessed in a phase III trial of patients with relapsed/refractory NSCLC following ≥ 1 line of chemotherapy, and whose tumors had progressed after disease control of ≥ 12 weeks with erlotinib or gefitinib, and thereafter, afatinib. The combination of afatinib with paclitaxel significantly improved tumor response and PFS compared with paclitaxel alone in patients who had EGFR tyrosine kinase inhibitor (TKI)-resistant (including afatinib) disease [19]. Conversely, in the phase III IMPRESS study, gefitinib plus cisplatin and pemetrexed in patients with *EGFR* mutation-positive advanced NSCLC and acquired resistance to first-line gefitinib did not prolong PFS in the overall population versus cisplatin plus pemetrexed [20]. Indeed, OS was inferior in the experimental arm of this trial, although there was a suggestion of improved outcomes in patients lacking the T790M resistance mutation [21].

In the current study, the safety and clinical activity of two new afatinib combinations in patients with advanced solid tumors are presented. The RP2Ds of oral afatinib in these new combinations were defined as 40 mg/day with carboplatin AUC6, and 20 mg/day with carboplatin AUC5 and paclitaxel 175 mg/m^2^. These regimens may be of potential interest for further study, for example, in patients with squamous NSCLC; in selected populations with *EGFR* mutations; and, particularly for the combination of afatinib and carboplatin, in elderly populations and patients with an ECOG PS of ≥ 2.
